# The sexual and reproductive health of women with mental illness: a primary care registry study

**DOI:** 10.1007/s00737-022-01214-y

**Published:** 2022-04-02

**Authors:** Holly Hope, Matthias Pierce, Edward D. Johnstone, Jenny Myers, Kathryn M. Abel

**Affiliations:** 1 Centre for Women’s Mental Health, Division of Psychology and Mental Health, Faculty of Biology Medicine and Health, University of Manchester, Room 3.320 Jean Mac Farlane Building, Oxford Road, Manchester, M13 9PL UK; 2 Maternal & Fetal Health Research Centre, Division of Developmental Biology & Medicine, Faculty of Biology, Medicine and Health, University of Manchester, Manchester, UK; 3 Manchester University Hospital NHS Foundation Trust, Manchester Academic Health Science Centre, Manchester, UK; 4 Greater Manchester Mental Health NHS Foundation Trust, Prestwich, Manchester, UK

**Keywords:** Mental illness, Reproductive health, Fertility, Sexual health, Contraception, Cervical screening

## Abstract

The purpose of this study is to characterise the sexual and reproductive health risks associated with mental illness among women. This was a retrospective cohort study of 2,680,149 women aged 14 to 45 years in the Clinical Practice Research Datalink, a UK primary care register, linked to 1,702,211 pregnancies that ended between the 1st January 1990 and 31st December 2017. Mental illness was identified in primary care and categorised into the following: common mental illness (depression/anxiety); addiction (alcohol/drug misuse); serious mental illness (affective/non-affective psychosis); other mental illness (eating/personality disorders). Logistic regression estimated the association between mental illness and subsequent risk of recurrent miscarriage and termination. Cox proportional hazards estimated the association between mental illness and time to gynaecological diseases, sexually transmitted infections, reproductive cancers, cervical screen, contraception and emergency contraception. Models were adjusted for calendar year, year of birth, smoking status and ethnicity, region and index of socioeconomic status. Compared to women without mental illness, exposed women were more likely to experience recurrent miscarriage (adjOR = 1.50, 95%CI 1.41 to 1.60), termination (adjOR = 1.48, 95%CI 1.45 to 1.50), gynaecological diseases (adjHR = 1.39, 95%CI 1.37 to 1.40), sexually transmitted infections (adjHR = 1.47, 95%CI 1.43 to 1.51), reproductive cancers (adjHR = 1.10, 95%CI 1.02 to 1.19), contraception (adjHR = 1.28 95%CI 1.26 to 1.29) and emergency contraception (adjHR = 2.30, 95%CI 2.26 to 2.34), and less likely to attend for cervical screening (adjHR = 0.91, 95%CI 0.90 to 0.92). Currently, the sexual and reproductive health needs of women with mental illness are unmet representing significant health inequalities. Clinicians must create opportunities to engage with women in primary care and mental health services to address this gap.

**Supplementary Information:**

The online version contains supplementary material available at 10.1007/s00737-022-01214-y.

Women aged between 20 and 40 years are seen 2.5 times more often than men in primary care, which is equivalent to 44.6 million additional healthcare visits per annum using 2020 population estimates (Wang et al. 2013); much of this excess is due to sexual and reproductive health. Sexual and reproductive health is fundamental to wellbeing and demonstrates clear overlap with universal human rights including the ability and opportunity to choose if and when to have children (WHO 2017). Like most women, women with mental illness are more likely than not to experience pregnancy and parenthood over their lifetime (Maybery and Reupert 2018). But women with mental illness are less fertile, particularly women with psychotic disorder (Hope et al. 2020; Pierce et al. 2020; Power et al. 2013a, b; Vigod et al. 2012). Potentially, a large amount of the NHS’s sexual and reproductive health need is among women with mental illness but, until now, we have lacked the necessary datasets to examine this.

Reproductive health in the context of mental illness is complex and is influenced by a broad range of medical, social and environmental factors. Reduced fertility is still seen in women following introduction of second-generation antipsychotics, and these are less liable to increase prolactin and, thereby, reduce fertility (Hope et al. 2020). Higher rates of smoking and obesity in women with mental illness may increase risk of polycystic ovarian syndrome, cancers and endometriosis (Saha et al. 2017). Women with mental illness may have less choice over their sexual and reproductive lives: they are more likely to experience coercive sex and rape (Abel and Rees 2010) which increases their risk of sexually transmitted diseases and associated fertility problems, and the need for emergency contraception (EC). Women with a history of domestic violence are also substantially more likely to experience poor mental health and to use EC (Jackson et al. 2019). Despite these concerns, little contemporary information is available to guide clinical practice and clinicians may continue to be unaware, or not to take account, of the sexual and reproductive health risks for women with mental illness.

Using a large UK primary care database, we investigated the association between mental illness and subsequent sexual and reproductive health outcomes. First, we hypothesised that women with mental illness would have increased risk of sexually transmitted infections, gynaecological diseases and reproductive cancers, compared with women without mental illness. Secondly, if women with mental illness are at higher risk reproductive cancers, then this cohort may also have fewer cervical screens. Thirdly, if women with mental illness have less choice over when they become pregnant, then their rate of prophylactic and emergency contraception use will be lower and higher than women without mental illness. Finally, if women with mental illness experienced poor reproductive health and lacked reproductive choice, then we hypothesised this cohort would experience recurrent miscarriage and undergo termination of pregnancy at a higher rate, compared to women without mental illness.

## Methods

### Design and data sources

This is a retrospective cohort study using a primary care database: the UK Clinical Practice Research Datalink (CPRD) GOLD. The CPRD contains individual-linked data on over 15 million patients, including data on clinical consultations, treatments, referrals and tests, in addition to patient demographics and practice data (Herrett et al. 2015).

We linked women to their pregnancies using the CPRD Pregnancy Register, which is an algorithm that calculates pregnancy start and end dates and outcomes (Minassian et al. 2019). We also linked to an area-level deprivation measure — the Index of Multiple Deprivation (IMD) dataset — using the location of each women’s general practice. This is currently available for 75% of English practices registered in CPRD GOLD (Kontopantelis et al. 2018).

### Study population

The cohort for this analysis was drawn from 3,624,708 women aged 14–44 years registered at a CPRD GOLD–participating practice from the 1st January 1990 to 31st December 2017. Data were extracted from the date when certain quality standard measures were met (Herrett et al. 2015). Eligible women were those registered for at least 2 years at a general practice, to establish prior exposure to mental illness. This resulted in 2,680,149 women in the analysis.

**Fig. 1:**
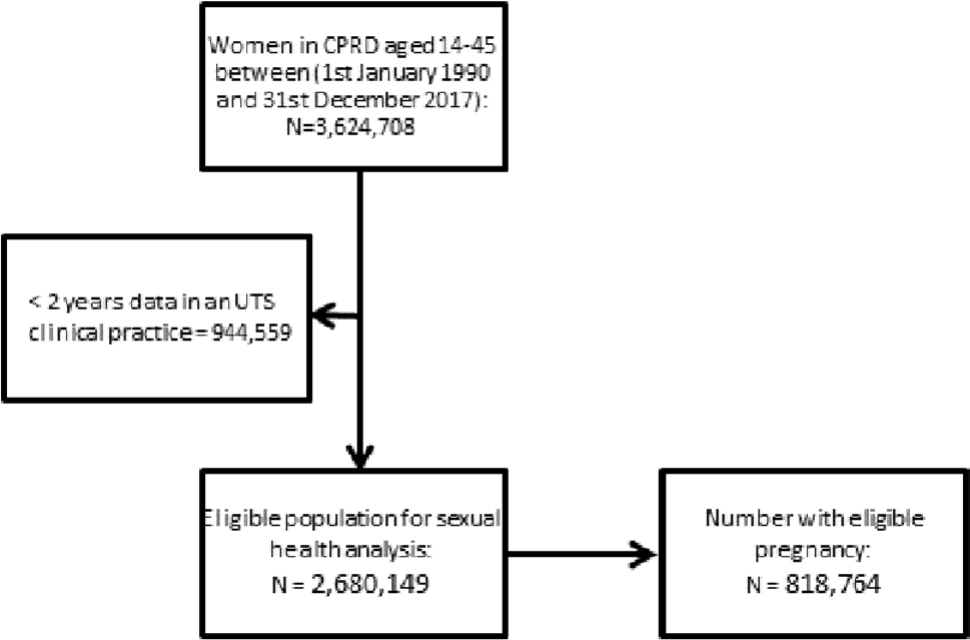
Diagram to show cohort selection

Follow-up began on the latest date of the following: 14th birthday; registration at a practice; CPRD date practice up to standard; and ended at the earliest date of: 45th birthday; study end date; CPRD transfer out date; date left clinical practice. After linkage to the Pregnancy Register, 818,764 women had at least one pregnancy during follow-up (see Fig. 1).

### Outcomes

Information on sexual and reproductive health during follow-up was extracted from GP medical records, a full list of the diseases is available in a supplementary document and the read codes used are available at clinicalcodes.org. These included:

1. Sexually transmitted infection (STI) including chlamydia, gonorrhoea, syphilis, genital warts, genital herpes and HIV/AIDS. HIV was also identified from prescriptions of HIV anti-viral medication.
2. Diagnoses of gynaecological diseases (inflammatory and non-inflammatory) such as primary or secondary infertility (ovulatory or tubal), polycystic ovarian syndrome, tubal disease, peritoneal disease, uterine abnormalities, thyroid abnormalities, endometriosis or fibroids and menstrual disorders (i.e. amenorrhoea, oligo menorrhoea, menorrhagia).
3. Cancers that affect a woman’s reproductive system, which include cervical, breast, ovarian, endometrial and vulval cancers.
4. Contacts for cervical screening and cervical smears after an abnormal test result.
5. Prescriptions for female prophylactic contraception (long-term, e.g. implant and intrauterine device, or contraceptive pill) and emergency contraception (EC; levonorgestrel or ulipristal acetate).
6. Recurrent miscarriage or termination of pregnancy. Recurrent miscarriage was defined as the third or higher consecutive miscarriage, (i.e. uninterrupted by a live birth) recorded in the pregnancy register.

### Exposure

Mental illness was categorised as follows: serious mental illness (affective or non-affective psychosis); common mental illness (depression or anxiety); addiction (substance or alcohol misuse disorder); and other mental illness (eating or personality disorder). Mental illness was identified using a previously developed algorithm that uses prescription, diagnosis and symptom data (Abel et al. 2019): a diagnosis was considered sufficient; however, a mental illness symptom (or prescription) required either a historical diagnosis or a prescription (or symptom) within 3 months.

### Other covariates

The following data were extracted from the women’s medical records: year of birth, smoking status (current/ex/never-smoked) and ethnicity (Asian/British Asian, Black/Black British, Mixed, Other and White ethnic groups). Unknown/missing ethnicity (63.5%) and missing smoking status (14.2%) were coded as a separate category. UK region of the general practice (Scotland, Wales and Northern Ireland) and region of England (North East, North West, Yorkshire & The Humber, East Midlands, West Midlands, East of England, South West, South Central, London and South East). The IMD score linked to women’s GP was ranked from lowest to highest and divided into quintiles, where the fifth quintile represents those areas of England that are most deprived. For this analysis, 21.1% of women were from practices outside England and were not assigned a rank.

### Analysis

The incidence of sexual or reproductive health outcomes were compared between women with and without mental illness using a time-to-event framework. For each analysis, women were censored on the date of the first date of a sexual or reproductive health outcome or the end of follow-up. Mental illness was treated as a time-dependent exposure. All events of common mental illness were captured and combined into discrete episodes, and a new episode began when there was a ≥ 2 year gap between events. In this analysis, women were exposed to a maximum number of seven episodes. For other mental illnesses, they were exposed from the date of the exposure until the end of follow-up.

The incidence of each outcome was compared between women with and without mental illness using Cox proportional hazard models, where survival time was measured in age. Adjusted hazard ratios were calculated by including variables in the model for women’s smoking status at the beginning of follow-up (ever current smoker, ever ex-smoker, never smoked), ethnicity and year of birth, and the region and level of deprivation of the general practice.

To investigate the association between mental illness and pregnancy outcome (recurrent miscarriage, termination), all pregnancies that occurred during follow-up were included; therefore, women could contribute more than one pregnancy to the analysis. Exposure to mental illness was indicated when women had a record of mental illness in the 2 years prior to the pregnancy start date. Logistic regression was used to estimate the association between mental illness and pregnancy outcomes. Huber-White sandwich estimators were used to calculate standard errors, taking account of the non-independence of pregnancies within women. Adjusted odds ratios included variables for age group (categorised, using 5-year intervals), ethnicity and smoking status of the woman, calendar period (categorised using 4-year intervals), the practice region and IMD.

## Results

### Sample description

The final sample consisted of 2,680,149 women with a total follow-up time of 14,069,182 person-years, and 529,843 (19.8%) were exposed to any mental illness during follow-up. Summary descriptive statistics reveal women with serious, addiction and other mental illness were younger than women with common mental illness or women without mental illness. More women with mental illness were more likely to smoke and tended to be registered at general practices situated in areas of higher socioeconomic deprivation (see Table 1).

**Table 1:** Characteristics of women across all mental illness groups

	None		Any		Common		Serious		Addiction		Other	
*N*	2,150,306		529,843		510,490		18,783		23,823		21,211	
Characteristic												
Year at start, median [IQR]	2003	[1999–2009]	2001	[1997–2005]	2001	[1997–2005]	2002	[1998–2007]	2001	[1997–2005]	2002	[1997–2005]
Age at start, median [IQR]	26.0	[33.5–44.5]	28.9	[35.6–44.5]	29.1	[21.6–35.6]	23.0	[15.8–35.7]	28. 0	[20.7–34.1]	19.7	[14.0–28.1]
Ethnicity	*N*	%	*N*	%	*N*	%	*N*	%	*N*	%	*N*	%
Asian/British Asian	56,166	2.6	5,272	1.0	4,946	1.0	410	2.2	93	0.4	181	0.9
Black/Black British	38,037	1.8	3,372	0.6	3,022	0.6	401	2.1	116	0.5	112	0.5
Mixed	12,123	0.6	1,875	0.4	1,754	0.3	130	0.7	80	0.3	104	0.5
Other	28,732	1.3	2,191	0.4	2,076	0.4	140	0.8	41	0.2	85	0.4
White	625,385	29.1	204,502	38.6	198,402	38.9	7,263	38.7	9,160	38.5	7,356	34.7
Unknown	1,389,863	64.6	312,631	59.0	300,290	58.8	10,439	55.6	14,333	60.2	13,373	63.1
Smoking status												
Never	1,238,729	57.6	261,379	49.3	252,633	49.5	8,084	43.0	4,838	20.3	11,272	53.1
Former	176,180	8.2	58,608	11.1	57,399	11.2	1,760	9.4	1,608	6.8	1,571	7.4
Current	374,767	17.4	190,741	36.0	183,543	36.0	8,119	43.2	16,031	67.3	6,762	31.9
Missing	360,630	16.8	19,115	3.6	16,915	3.3	820	4.4	1,346	5.7	1,606	7.6
Index of Multiple Deprivation												
1	294,222	17.1	60,624	15.3	58,432	15.3	1,988	14.0	1,808	11.0	2,654	16.2
2	316,312	18.4	69,230	17.4	66,610	17.4	2,256	15.9	2,515	15.3	3,044	18.6
3	341,018	19.8	78,228	19.7	75,498	19.7	2,680	18.9	2,902	17.7	3,126	19.1
4	375,995	21.9	82,772	20.8	79,541	20.8	3,226	22.7	3,403	20.8	3,315	20.2
5	391,485	22.8	106,723	26.8	102,493	26.8	4,070	28.6	5,773	35.2	4,268	26.0

### Sexually transmitted infections (STIs)

There was an increased risk of STIs associated with mental illness (rate = 5.77 versus 4.43, HR = 1.47, 95%CI 1.43–1.45). Women with addiction disorder (rate = 6.94, adjHR = 1.56, 95%CI 1.42–1.71) or other mental illness (rate = 8.24, adjHR = 1.46, 95%CI 1.35–1.58) were at the highest risk of a sexually transmitted infection, compared to unexposed women.

### Gynaecological diseases

**Fig. 2:**
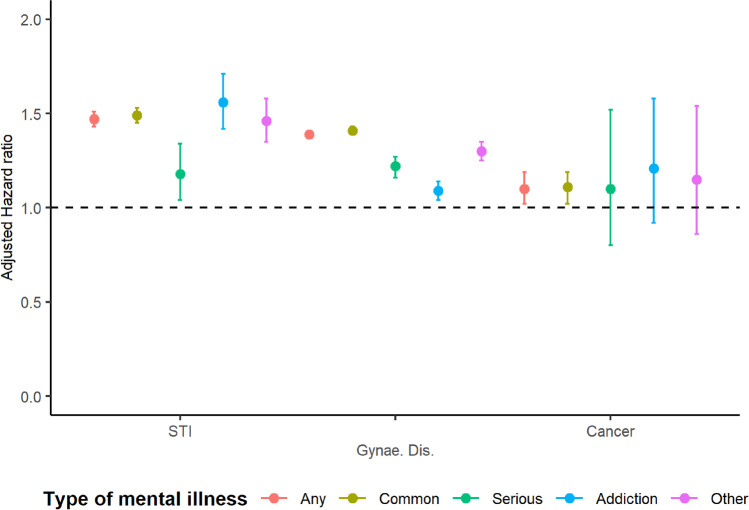
The effect of specific types of mental illness on the risk of reproductive diseases adjusted for adjusted for ethnicity, year of birth, region of the UK and Index of Multiple Deprivation quintile

Compared to women without mental illness, women with mental illness had increased rates of gynaecological diseases; this increase remained after adjustment for all covariates (rate = 55.6 versus 40.4 per 1000 person-years, adjHR = 1.39, 95%CI 1.37–1.40). Women with serious mental illness, addiction disorders and other mental illness were also at increased risk of gynaecological diseases compared to unexposed women (eTable 1, Fig. 2).

### Cancer

The rate (per 1000 person-years) of cancers of the reproductive system was higher for women with mental illness (0.65) than women without mental illness (0.43). After adjustment for covariates, there was an 11% increase in the risk of cancers of the reproductive system (aHR = 1.11; 95%CI 1.03–1.19). Rates of cancers were similar for women across mental illness groups; however, only the hazard ratio comparing women with common mental illness and no mental illness was conclusive (adjHR = 1.11, 95%CI 1.02–1.19).

### Reproductive health contacts

The crude rates (per 1000 person-years) of cervical health contacts for other mental illness (eating and personality disorders) (38.2) was lower than women without mental illness (44.8). Women with common (51.2), serious (46.2) and addiction (46.2) had a higher rate of contacts for cervical health; however, after adjustment for age and other covariates, there was a lower hazard compared to unexposed women (common mental illness adjHR = 0.92, 95%CI 0.90–0.93).

Contraception use varied by type of mental illness. Compared to unexposed women, women with common mental illness were more likely to receive a contraception prescription in primary care (rate = 99.4 versus 105.7, adjHR = 1.28, 95%CI 1.26–1.29), whilst women with serious mental illness (rate = 65.0, adjHR = 0.90, 95%CI = 0.86–0.94) and addiction (adjHR = 0.86, 95%CI 0.82–0.90) were less likely.

**Fig. 3:**
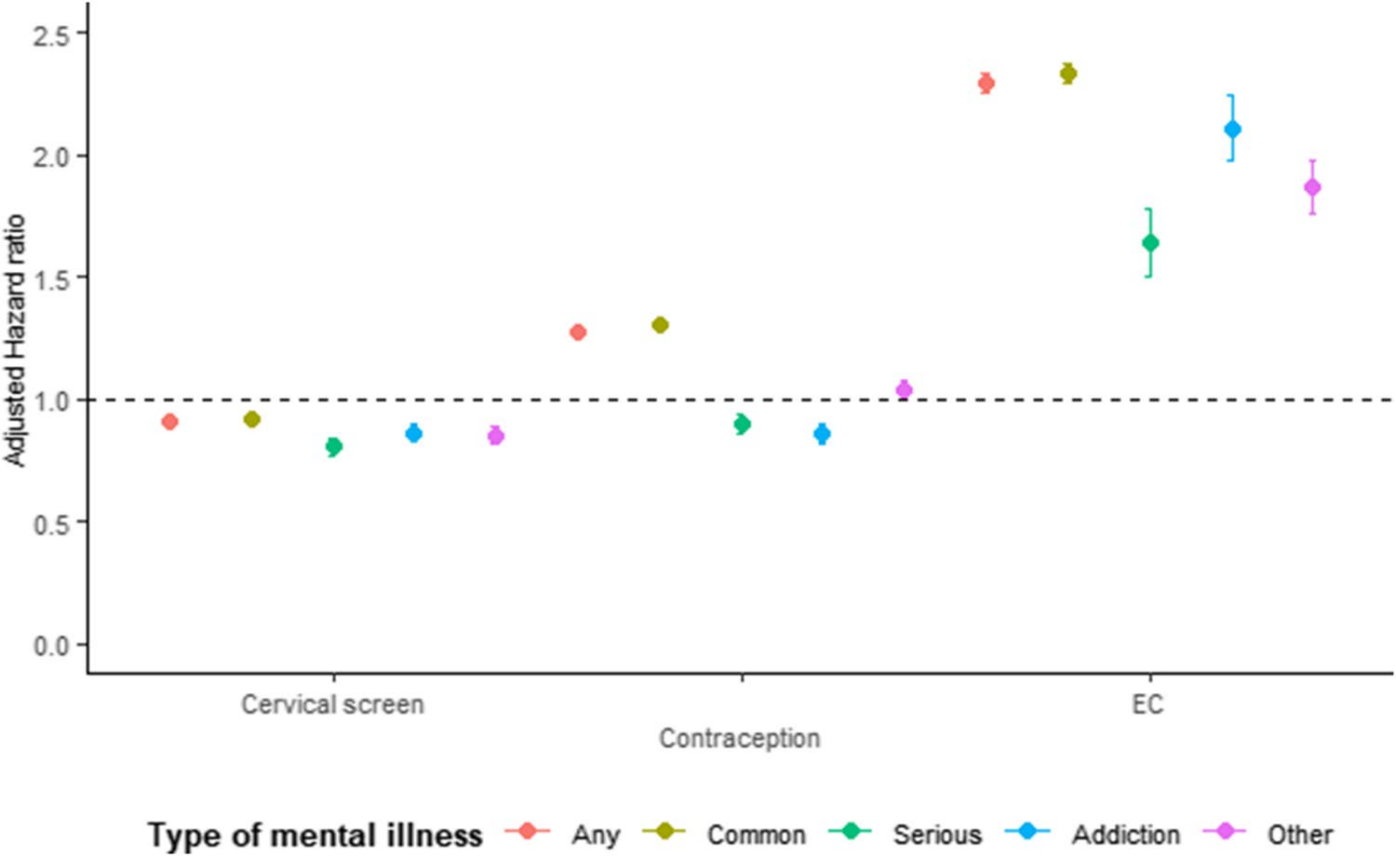
The effect of specific types of mental illness on the risk of attending primary care for cervical screening, prophylactic contraception or emergency contraception (EC) adjusted for ethnicity, year of birth, region of the UK and Index of Multiple Deprivation quintile

The rate of emergency contraception use among women with mental illness (13.8) was double that of women without mental illness (6.7; adjHR = 2.30, 95%CI 2.26–2.34) (eTable2, Fig. 3).

### Recurrent miscarriage or termination of pregnancy

**Fig. 4:**
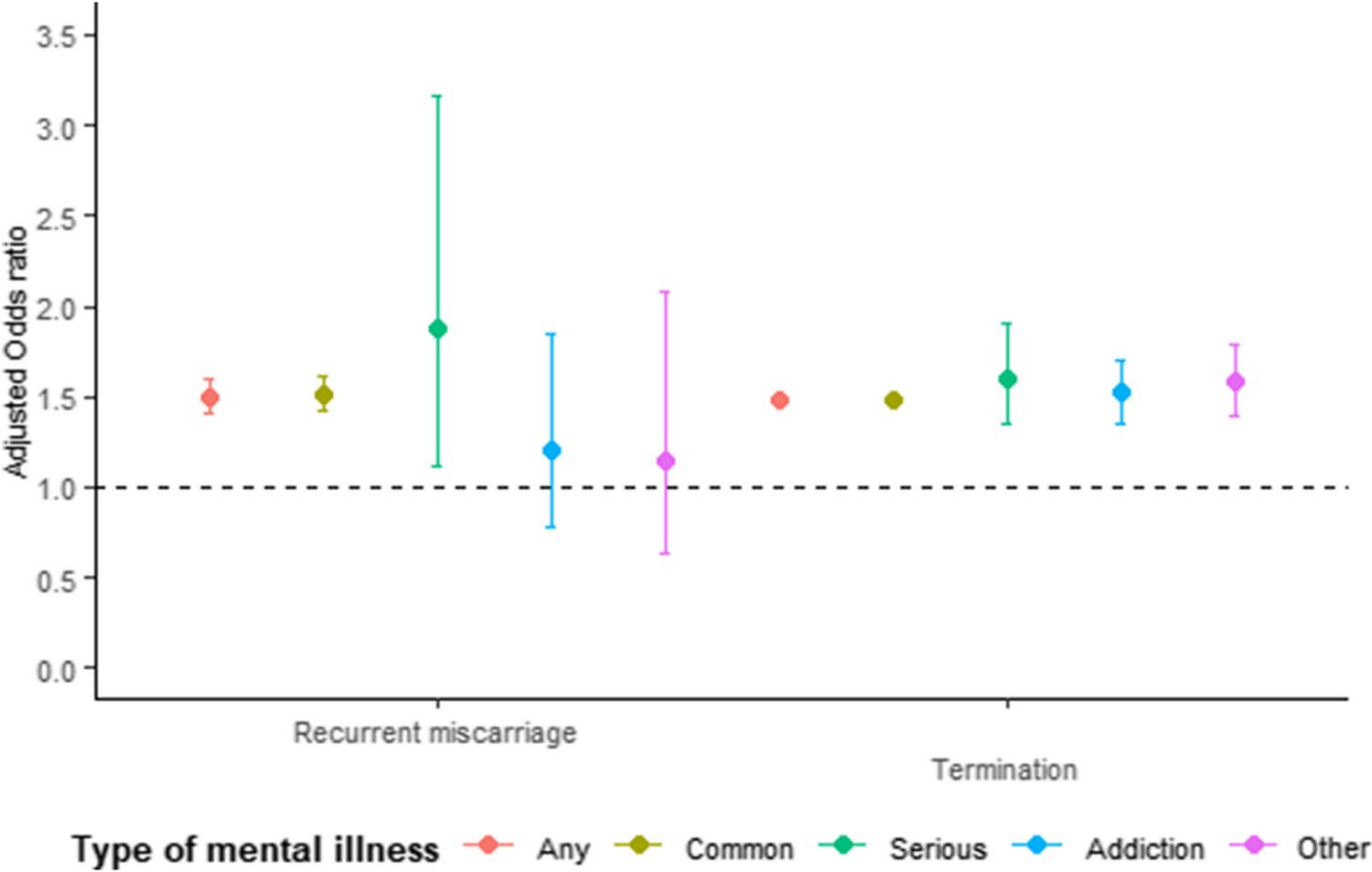
The effect of specific types of mental illness on the risk of recurrent miscarriage or termination, adjusted for adjusted for age, ethnicity, smoking status, calendar period, region of the UK and Index of Multiple Deprivation quintile

There were 1,702,211 pregnancies during follow-up. After adjusting for age, smoking, ethnicity and IMD, women with any mental illness were 50% more likely to experience recurrent miscarriage (1.1% versus 0.7%, adjOR 1.50, 95%CI 1.41–1.60) compared to women without mental illness. The association was greater for women with serious mental illness (adjOR = 1.87, 95%CI 1.11–3.17). Higher proportions of pregnancies ended in termination among women with mental illness (12.62 versus 9.1%, adjOR 1.48, 95%CI 1.35–1.50), serious mental illness (14.2%, adjOR 1.64, 95%CI 1.11–3.177), other mental illnesses (eating and personality disorders; 17.5%, adjOR 1.57, 95%CI 1.40–1.79) and addiction (17.4%, adjOR 1.52, 95%CI 1.35–1.70) (eTable 1, Fig. 4).

## Discussion

In this large cohort of contemporary UK women of reproductive age, we show a general pattern of sexual and reproductive health inequity for women with mental illness compared to women without. They are more likely to experience an increased risk of sexually transmitted infections, gynaecological diseases and reproductive health cancers, all of which may affect fertility and pregnancy outcomes. Despite this, women with mental illness are less likely to access cervical screening than well women, whilst their risk of receiving emergency, as opposed to prophylactic, contraception was double that of women in the general population. This is consistent with our other finding that women with mental illness were 50% more likely to experience recurrent miscarriage and twice as likely to undergo a termination and represents a clear narrative of missed opportunities to improve outcomes.

Some types of mental illness were associated with greater risks. Women with addiction disorders, for example, were at the highest risk of STIs and cancers. Terminations were also significantly more; and contraception use significantly less frequent in women with an addiction, serious and other mental illness, compared to the rest of the population. Finally, women with common mental illness were more and women with serious, addiction and other mental illness less likely to access prophylactic contraception, compared to the rest of the population.

### Research in context

Prior work has identified that women with bipolar disorder, addiction and other serious mental illnesses are more likely to undertake risky sexual behaviour such as unprotected intercourse and to have multiple partners (Marengo et al. 2015; Meade and Sikkema 2005). We report that women with depression or anxiety are also at risk of sexually transmitted diseases and experience higher need for emergency contraception (Abel et al. 2010). This supports the notion that, overall, women with mental illness are less likely to have a choice over their reproductive life and that rape, intimate partner violence and coercive control may have direct consequences for women’s health apart from her mental health.

Our findings extend previous research into the link between mental illness and fertility and provide the sexual and reproductive health context of women with mental illness (Hope et al. 2020; Pierce et al. 2020; Power et al. 2013a, b). This is likely to be an area where clinical awareness remains limited (Abel and Rees 2010). Moreover, women with serious mental illness and addiction disorders are less likely to access family planning, but are also experiencing reduced fertility relative to other women (Hope et al. 2020), which signals significant unmet reproductive health needs in this group. The increased risk of miscarriage and termination among women with mental illness replicates other’s work (Laursen and Munk-Olsen, 2010; Linna et al. 2013; Magnus et al. 2021); however, to date, this is the first report of the rarer outcome of recurrent miscarriage: a clear indication of poor reproductive health. These effects are independent of smoking status, which suggests interventions need to address other factors alongside smoking cessation.

### Clinical and policy impact

The current NHS long-term plan aims to develop maternity outreach clinics that will integrate psychological therapies within maternity services and improve specialist perinatal mental health services. Furthermore, the physical health of people with mental illness is now a priority for NHSE (Mental Health Task force 2016). Notwithstanding, neither initiatives include sexual or reproductive health of people with mental illness. In 2011, DHSC funded open-access, online training and educational resources aimed at mental health nurses and women (SCIE n.d.) which do address the overlap between sexual, reproductive and mental health. More recently, the charity ‘Tommy’s’ (Tommys.org.uk) launched their web-based information for perinatal mental health and included limited preconception advice.

However, the sexual and reproductive health needs of women with mental illness are not a focus for clinicians in primary or secondary care (Abel and Rees 2010) and there remains a pressing need to develop broader clinical awareness and create opportunities to engage with women with mental illness about these aspects of their health. Conversations about sexual choice, safety, preferred method of contraception and family planning are needed as part of routine care, as well as checking cervical screening or HPV vaccination uptake. General practitioners and mental health clinicians require training in the skills to talk to women about their sexual and reproductive health and to support women to make choices that protect this alongside their mental health needs. There is increasing evidence that midwife‐led continuity of carer systems improves pregnancy and infant outcomes among at-risk groups (Sandall et al. 2016). If such models were extended to include pre-conception care pathways, women would have a named person who understands their specific sexual and reproductive health needs.

### Limitations

We used survival analysis, where the denominator is person-time rather than population to minimise biases because of variation in follow-up. We did not left censor; this approach opens the possibility of reverse causality; therefore, post hoc we censored women with a record of the outcome prior to the start of follow-up and the results were similar (not presented). The aim of this study was to provide explanation(s) for why women of reproductive age with mental illness experience poorer reproductive health; except for cervical cancer, cancers mainly affect women over the age of 45 years, meaning this study does not capture the true reproductive cancer burden associated with mental illness. Primary care data will not contain all contraception and emergency contraception use as women in the UK may access over-the-counter contraception or use sexual health services for family planning.

### Suggested mechanisms and future research

The robust association between mental illness and reproductive health suggest shared genetic and environmental mechanisms. For example, polycystic ovarian syndrome consistently associates with all types of mental illness, independent of psychotherapeutics, which might indicate a role for androgen-mediated mechanisms (Cesta et al. 2016). The extent to which the increased risk of sexually transmitted infections and gynaecological disease might mediate the association between mental illness and fertility, separately from shared genetic and environmental mechanisms (e.g. poverty, obesity, smoking, poor diet and exercise), is deserving of further research. Similarly, the association between mental illness and gynaecological disease, specifically disease of the pelvis and fallopian tube, is likely to be least partly mediated by the increased risk of sexually transmitted diseases. Research that aims to delineate and quantify the direct and indirect pathways linking mental illness and reproductive health with a focus on the modifiable mediators of risk might demonstrate how to reduce such multimorbidity over the life course, where women with mental illness are over-represented (Lee et al. 2021). The reported reproductive health effects associated with mental illness are adjusted for important covariates such as age, deprivation and ethnicity. These covariates may also moderate the association between mental illness and reproductive health and future analyses should explore if such effect modifications exist to identify groups of women for whom preventive interventions might be of particular benefit. Acceptability and feasibility studies of tailored and targeted strategies in healthcare settings to prevent poor sexual and reproductive health among women with mental illness are now a priority.

## Conclusion

We demonstrate significant inequity in the sexual and reproductive health of women with mental illness living in the UK today affecting their human reproductive rights. Clinical training and awareness should be prioritised which might achieve considerable change with little additional resource.

## Supplementary Information

Below is the link to the electronic supplementary material.

Supplementary file1 (DOCX 15 KB)

Supplementary file2 (DOCX 21 KB)

Supplementary file3 (DOCX 25 KB)

## Data Availability

Read codes used are published on clinicalcodes.org. Electronic health records are, by definition, considered ‘sensitive’ data in the UK by the Data Protection Act 2018 and cannot be shared via public deposition because of information governance restriction in place to protect patient confidentiality. Access to data is available only once approval has been obtained through the individual constituent entities controlling access to the data. The primary care data can be requested via application to the Clinical Practice Research Datalink (www.cprd.com/researcher); secondary care data can be requested via application to the Hospital Episode Statistics from the UK Health and Social Care Information Centre (www.hscic.gov.uk/hesdata).

## Code availability

The dofiles used in the Stata software to analyse the data are available from the corresponding author on request.
