# Supplementary material for: The sexual and reproductive health of women with mental illness: a primary care registry study
Source: Arch Womens Ment Health. 2022 Apr 2;25(3):585–93. doi: 10.1007/s00737-022-01214-y (PMC9072520; doi:10.1007/s00737-022-01214-y)
Supplement: Supplementary file 1 — Supplementary file1 (DOCX 15 KB) [file 737_2022_1214_MOESM1_ESM.docx]

Supplementary methods

We extracted the dates for the following sexual and reproductive health outcomes for all women in the cohort utilising primary care records.

1. Sexually transmitted infections (STI)
   1. Chlamydia, gonorrhoea, syphilis, genital warts, genital herpes, HIV/Aids, endometritis, STD NOS.
   2. HIV was defined as earliest date of HIV infection, AIDS diagnosis or HIV anti-viral medication (i.e. prescription of a drug listed in Chapter five section three sub-paragraph one of the British National Formulary (BNF).
2. Gynaecological diseases
   1. Ovulatory dysfunction (i.e. Primary/secondary anovulatory infertility, polycystic ovaries, infertility of pituitary - hypothalamic origin, which includes Sheehan's syndrome, Pituitary dependent Cushing's syndrome, Stein - Leventhal syndrome, Kallman's syndrome, Malignant neoplasm of pituitary gland), Tubal disease (i.e. Primary/Secondary tubal infertility, Blocked fallopian tube), Polycystic Ovarian Syndrome, Peritoneal disease (i.e. Peritoneal adhesions, Tubo-ovarian peritoneal adhesions), Uterine abnormalities (i.e. Primary/secondary infertility of cervical or uterine origin, cervical infertility metritis, endometritis, bicornate uterus, uterine adhesions), Thyroid abnormalities (i.e. hypo/hyperthyroidism), Primary/secondary infertility (NOS).
   2. Menstrual Health: Amenorrhoea/ Oligomenorrhoea, Pelvic pain (i.e. dyspareunia, dysmenorrhea, adenomyosis, chronic pelvic pain), Pelvic inflammatory disease, Menorrhagia, Endometriosis, Fibroids.
3. Cancers associated with reproduction:
   1. Cervical cancer, Breast cancer, Ovarian cancer, Endometrial cancer, Vulval cancer
4. Reproductive healthcare:
   1. Cervical screening: routine cervical smears, Pap. smears, Human Papilloma Virus test (but not cervical/vaginal swabs) and cervical smears after an abnormal test result.
   2. Contraception: Includes every prescription for a long-term, short-term, or contraceptive pill.
   3. Emergency contraception: Any prescription for emergency hormonal contraception (EHC) (Levonorgestrel or Ulipristal Acetate) or primary care contact to request emergency contraception (see clinicalcodes.org).
