# Supplementary material for: The sexual and reproductive health of women with mental illness: a primary care registry study
Source: Arch Womens Ment Health. 2022 Apr 2;25(3):585–93. doi: 10.1007/s00737-022-01214-y (PMC9072520; doi:10.1007/s00737-022-01214-y)
Supplement: Supplementary file 2 — Supplementary file2 (DOCX 21 KB) [file 737_2022_1214_MOESM2_ESM.docx]

eTable 1 Table displaying rates (per 1000 person-years) and hazard ratios of reproductive health outcomes for women across mental illness categories

|  |  | Exposure to mental illness | | | | | | | | | | | |
| --- | --- | --- | --- | --- | --- | --- | --- | --- | --- | --- | --- | --- | --- |
| Outcome | Statistic | None | | Any | | Common | | Serious | | Addiction | | Other | |
| STI | Rate (95%CI) | 4.43 | (4.40-4.47) | 5.77 | (5.66-5.88) | 5.73 | (5.61-5.84) | 4.40 | (3.96-4.89) | 6.94 | (6.44-7.48) | 8.24 | (7.70-8.82) |
|  | HR (95%CI) |  |  | 1.63 | (1.59-1.66) | 1.65 | (1.61-1.68) | 1.37 | (1.23-1.52) | 1.89 | (1.76-2.04) | 1.57 | (1.46-1.68) |
|  | adjHR (95%CI) |  |  | 1.47 | (1.43-1.51) | 1.49 | (1.45-1.53) | 1.18 | (1.04-1.34) | 1.56 | (1.42-1.71) | 1.46 | (1.35-1.58) |
| Gynae. Dis. | Rate (95%CI) | 40.43 | (40.32-40.54) | 55.61 | (55.19-56.04) | 56.53 | (56.09-56.98) | 51.59 | (49.67-53.57) | 45.84 | (44.24-47.50) | 55.20 | (53.41-57.05) |
|  | HR (95%CI) |  |  | 1.42 | (1.41-1.44) | 1.45 | (1.44-1.46) | 1.28 | (1.23-1.33) | 1.13 | (1.09-1.17) | 1.31 | (1.27-1.36) |
|  | adjHR (95%CI) |  |  | 1.39 | (1.37-1.40) | 1.41 | (1.40-1.43) | 1.22 | (1.16-1.27) | 1.09 | (1.04-1.14) | 1.30 | (1.25-1.35) |
| Cancer | Rate (95%CI) | 0.44 | (0.43-0.45) | 0.66 | (0.63-0.70) | 0.67 | (0.63-0.71) | 0.65 | (0.50-0.85) | 0.65 | (0.51-0.82) | 0.68 | (0.54-0.86) |
|  | HR (95%CI) |  |  | 1.30 | (1.23-1.39) | 1.31 | (1.23-1.39) | 1.20 | (0.92-1.57) | 1.23 | (0.97-1.56) | 1.52 | (1.21-1.91) |
|  | adjHR (95%CI) |  |  | 1.10 | (1.02-1.19) | 1.11 | (1.02-1.19) | 1.10 | (0.80-1.52) | 1.21 | (0.92-1.58) | 1.15 | (0.86-1.54) |
| Cervical screen | Rate (95%CI) | 44.82 | (44.71-44.93) | 50.16 | (49.78-50.54) | 51.23 | (50.84-51.63) | 46.17 | (44.48-47.93) | 46.24 | (44.73-47.80) | 38.21 | (36.90-39.56) |
|  | HR (95%CI) |  |  | 0.96 | (0.96-0.97) | 0.97 | (0.97-0.98) | 0.87 | (0.83-0.90) | 0.89 | (0.86-0.92) | 0.90 | (0.87-0.93) |
|  | adjHR (95%CI) |  |  | 0.91 | (0.90-0.92) | 0.92 | (0.91-0.93) | 0.81 | (0.77-0.84) | 0.86 | (0.83-0.90) | 0.85 | (0.82-0.89) |
| Contraception | Rate (95%CI) | 105.69 | (105.49-105.89) | 99.36 | (98.66-100.07) | 99.45 | (98.72-100.19) | 65.00 | (62.66-67.42) | 67.87 | (65.56-70.26) | 116.21 | (112.88-119.63) |
|  | HR (95%CI) |  |  | 1.32 | (1.31-1.33) | 1.36 | (1.35-1.37) | 0.89 | (0.86-0.93) | 0.87 | (0.83-0.90) | 1.05 | (1.02-1.08) |
|  | adjHR (95%CI) |  |  | 1.28 | (1.26-1.29) | 1.31 | (1.29-1.32) | 0.90 | (0.86-0.94) | 0.86 | (0.82-0.90) | 1.04 | (1.00-1.08) |
| EC | Rate (95%CI) | 6.70 | (6.66-6.74) | 13.78 | (13.60-13.96) | 13.84 | (13.66-14.03) | 9.87 | (9.19-10.61) | 15.87 | (15.08-16.71) | 16.00 | (15.22-16.82) |
|  | HR (95%CI) |  |  | 2.60 | (2.56-2.64) | 2.65 | (2.61-2.69) | 1.82 | (1.70-1.96) | 2.66 | (2.53-2.81) | 1.99 | (1.89-2.09) |
|  | adjHR (95%CI) |  |  | 2.30 | (2.26-2.34) | 2.34 | (2.30-2.38) | 1.64 | (1.50-1.78) | 2.11 | (1.98-2.25) | 1.87 | (1.76-1.98) |

HR: Hazard Ratio; adjHR: Adjusted Hazard ratio; adjHR adjusted for age, ethnicity, smoking status, calendar period, region of the UK and Index of Multiple Deprivation quintile.

eTable 2 Table displays the odds of recurrent miscarriage and termination for women within each mental illness category

|  |  | Exposure | | | | | | | | | | | |
| --- | --- | --- | --- | --- | --- | --- | --- | --- | --- | --- | --- | --- | --- |
| Outcome | Stat | None(1,667,808) | | Any (230,186) | | Common | | Serious (N=1,802) | | Addiction (3,736) | | Other (2,939) | |
| Recurrent miscarriage | N (%) | 9,348 | (0.64) | 2,429 | (1.06) | 2,415 | (1.06) | 23 | (1.28) | 32 | (0.86) | 20 | (0.68) |
|  | OR (95%CI) |  |  | 1.64 | (1.55-1.73) | 1.65 | (1.56-1.74) | 1.70 | (1.04-2.79) | 1.42 | (0.97-2.08) | 1.40 | (0.85-2.31) |
|  | adjOR (95%CI) |  |  | 1.50 | (1.41-1.60) | 1.51 | (1.42-1.42) | 1.87 | (1.11-3.16) | 1.20 | (0.78-1.85) | 1.15 | (0.63-2.08) |
| Termination | % (95%CI) | 134,320 | (9.12) | 29,057 | (12.62) | 28,513 | (12.56 | 256 | (14.21) | 651 | (17.43) | 515 | (17.52) |
|  | OR (95%CI) |  |  | 1.48 | (1.46-1.50) | 1.48 | (1.46-1.50) | 1.64 | (1.41-1.90) | 1.71 | (1.55-1.88) | 1.57 | (1.41-1.75) |
|  | adjOR (95%CI) |  |  | 1.48 | (1.45-1.50) | 1.48 | (1.45-1.50) | 1.60 | (1.35-1.90) | 1.52 | (1.35-1.70) | 1.58 | (1.40-1.79) |

OR: Odds Ratio; adjHR: Adjusted Odds Ratio; adjOR adjusted for age, ethnicity, smoking status, calendar period, region of the UK and Index of Multiple Deprivation quintile.
